# Anti-depressant and anxiolytic like behaviors in PKCI/HINT1 knockout mice associated with elevated plasma corticosterone level

**DOI:** 10.1186/1471-2202-10-132

**Published:** 2009-11-13

**Authors:** Elisabeth Barbier, Jia Bei Wang

**Affiliations:** 1 Department of Pharmaceutical Sciences, School of Pharmacy, University of Maryland, 20 Penn Street, Baltimore, Maryland 21201, USA

## Abstract

**Background:**

Protein kinase C interacting protein (PKCI/HINT1) is a small protein belonging to the histidine triad (HIT) family proteins. Its brain immunoreactivity is located in neurons and neuronal processes. PKCI/HINT1 gene knockout (KO) mice display hyper-locomotion in response to D-amphetamine which is considered a positive symptom of schizophrenia in animal models. *Postmortem *studies identified PKCI/HINT1 as a candidate molecule for schizophrenia and bipolar disorder. We investigated the hypothesis that the PKCI/HINT1 gene may play an important role in regulating mood function in the CNS. We submitted PKCI/HINT1 KO mice and their wild type (WT) littermates to behavioral tests used to study anti-depressant, anxiety like behaviors, and goal-oriented behavior. Additionally, as many mood disorders coincide with modifications of hypothalamic-pituitary-adrenal (HPA) axis function, we assessed the HPA activity through measurement of plasma corticosterone levels.

**Results:**

Compared to the WT controls, KO mice exhibited less immobility in the forced swim (FST) and the tail suspension (TST) tests. Activity in the TST tended to be attenuated by acute treatment with valproate at 300 mg/kg in KO mice. The PKCI/HINT1 KO mice presented less thigmotaxis in the Morris water maze and spent progressively more time in the lit compartment in the light/dark test. In a place navigation task, KO mice exhibited enhanced acquisition and retention. Furthermore, the afternoon basal plasma corticosterone level in PKCI/HINT1 KO mice was significantly higher than in the WT.

**Conclusion:**

PKCI/HINT1 KO mice displayed a phenotype of behavioral and endocrine features which indicate changes of mood function, including anxiolytic-like and anti-depressant like behaviors, in conjunction with an elevated corticosterone level in plasma. These results suggest that the PKCI/HINT 1 gene could be important for the mood regulation function in the CNS.

## Background

Mood disorders, including depressive and bipolar disorders, are severe, chronic and often life-threatening illnesses with symptoms manifested at the psychological, behavioral, and physiological levels. Their etiology is not precisely understood but is believed to be multifactorial, involving heredity, changes in neurotransmitter levels, altered neuroendocrine function, and psychosocial factors [1]. In the laboratory, the biological study of mood disorders benefits from standardized animal behavioral tests that have been validated for their predictive validity for antidepressant and anxiolytic drugs. These tests are currently considered major tools for studying the function of targeted mutation in transgenic mice [2]. The forced swim (FST) and tail suspension (TST) were developed to evaluate anti-depressant like effects of drugs [3,4]. In genetically modified mice, anti-depressant-like behavior is modeled as a decreased immobility in the FST and the TST [5,6]. In comparison, the cyclic episodes of depression and mania characteristic of bipolar disorder is more difficult to model in a single animal. Therefore facets of bipolar disorder are commonly studied separately using distinct models for depression and mania [7,8]. However, in contrast to current models of depression-related behavior mentioned above, there is no specific model for studying mania-like behavior. Nonetheless, some paradigms are used for their similarity with clinical observations and are validated through their response to classical mood stabilizing drugs. For example, increased energy or restlessness can be modeled by the hyperactivity induced by psychostimulants, or as a response to a stressful situation and monitored in an open field test, and in the test related to a stressful situation [7]. Also, poor judgment leading to risk-taking can be tested as a mirror image of most tests for anxiety-like behavior [9]. Thus the light/dark paradigm based on the mice's natural avoidance of bright spaces is widely used to measure anxiety-like behavior in mutant mice [10]; animals that spend less time in the lit compartment are considered to exhibit anxiety-related behavior [11,12]. Similarly, open spaces in the open-field and in the Morris water maze serve as another index of anxiety-like behavior based on the propensity of small rodents to avoid open areas. In these paradigms anxiety-related behavior is assessed through the measure of thigmotaxis or wall-seeking behavior [13]. Furthermore, genetic vulnerability to mania is believed to be expressed behaviorally as excessive goal pursuit [14] which can be modeled using goal-oriented behavioral tests.

Pharmacological validation of mania-like behavior can be performed with valproate, a mood stabilizing agent used clinically to treat acute mania [15], which also decreases the phenotype of hyperactivity in the mouse model of mania [16].

In terms of endocrinology, mood disorders coincide with modifications of hypothalamic-pituitary-adrenal (HPA) axis function which can be assessed by measuring plasma glucocorticoid levels. A high plasma cortisol level is considered an endocrine feature of depression and bipolar disorder in patients [17-19]. Manic episodes have been shown to be preceded by increases in plasma cortisol level. Therefore HPA axis dysfunction was proposed as a potential trait marker in bipolar disorder, possibly indicative of the core pathophysiologic process in the illness [20]. In rodents, behavioral responses described as depression-like and anxiety-related coincide with increases in the level of plasma corticosterone [21-24]. In contrast, reduced depression-like and less anxiety-related behaviors are associated with a lowered level of plasma corticosterone [25,26]. In the animal models, therefore, increases in plasma corticosterone levels might argue for face validity at the endocrine level.

PKCI/HINT1 is a member of the HIT protein family, characterized by the presence of a HIT (HisXHisXHis, where X is a hydrophobic amino acid) sequence motif [27,28]. Although the general function of PKCI/HINT1, as well as its presumably more specialized role in the central nervous system, is not known, results from human *postmortem *analysis and mice behavioral and anatomical studies suggest that it might be involved in the pathophysiology of certain mental disorders. PKCI/HINT1 was identified as a candidate molecule in the neuropathology of schizophrenia when its gene expression was shown to be decreased *via *microarray analysis in the prefrontal cortex of schizophrenic patients [29,30], as subsequently validated by real-time quantitative polymerase chain reaction [31]. PKCI/HINT1 was also recognized as a gene candidate in the neuropathology of bipolar disorder when its decreased expression in the dorsolateral prefrontal cortex was confirmed in a meta-analysis of 12 microarray studies of bipolar disorder [32]. We recently showed that, although they do not show any spontaneous hyperactivity in the open field test, PKCI/HINT1 KO mice present higher locomotor and stereotypic responses to the psychostimulant D-amphetamine than WT controls. These are considered positive symptoms in animal models for schizophrenia [33]. Our study of the brain's distribution of PKCI/HINT1 revealed that its immunoreactivity is primarily located in neurons and neuronal processes of parvalbumin positive neurons in cerebral cortex and limbic system [34]. These regions are anatomically related to mood disorders [35-37].

Based on the findings from postmortem studies, PKCI/HINT1 KO mice behavioral observations, and immunocytochemical localization of the PKCI/HINT1 in the brain, we hypothesize that the PKCI/HINT1 protein might be involved in specific neuropsychological functions such that its absence would lead to significant behavioral and endocrine phenotypes associated with mood dysfunction. Accordingly, we attempted to test this hypothesis using PKCI/HINT1 KO mice. We assessed behavioral phenotypes that may be characteristic of mood disorders by using the forced swim and tail suspension tests, which are used to model antidepressant-like behavior. We also looked at the involvement of PKCI/HINT1 in the light/dark paradigm and in thigmotaxis as indices of anxiety-related behavior, and we used the Morris water maze test for place navigation learning to assess goal-oriented behavior. In order to search for associated endocrine relevance, we assessed the HPA axis activity through the measurement of plasma corticosterone levels. Pharmacologically, we examined the effect of valproate on the activity of the animals submitted to the tail suspension test.

## Results

### PKCI/HINT1 KO mice exhibited less immobility than their WT littermates in the forced swim and the tail suspension tests

**Figure 1:**
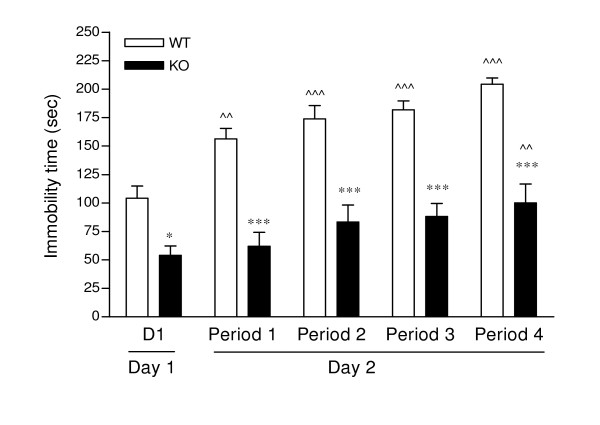
**Porsolt's forced swim test**. Scores of immobility in sec are represented for the last 4 min of each trial. ^^ P < 0.01, ^^^P < 0.0001 *vs*. Day1; * P < 0.05, *** P < 0.0001 WT *vs*. KO, Bonferroni *post hoc*. WT n = 15, KO n = 16.

Immobility in the forced swim test is shown in Figure 1, where the PKCI/HINT1 KO mice were 2 times less immobile than their WT littermates at every stage of the test from day 1 to each of the trials in the four time periods of the second day, F_(1,145) _= 145.55, P < 0.0001 for genotype in a two-way ANOVA of trials × genotype. Immobility time on day 1 represented 43% of the total time in the WT mice and 23% in the KO. When considering the four trials of the second day both WT and KO mice displayed increases in immobility over the trials, F_(4,145) _= 12.12, P < 0.0001 for trials effect, with similar slopes, F_(1,151) _= 0.519, P < 0.472. WT mice displayed a significantly progressive increase in immobility over the number of trials as compared with day 1; on the last trial, immobility time reached 85% of total time. KO mice showed less increase in immobility compared to WT on the last trial, with immobility reaching 42% of total time.

**Figure 2:**
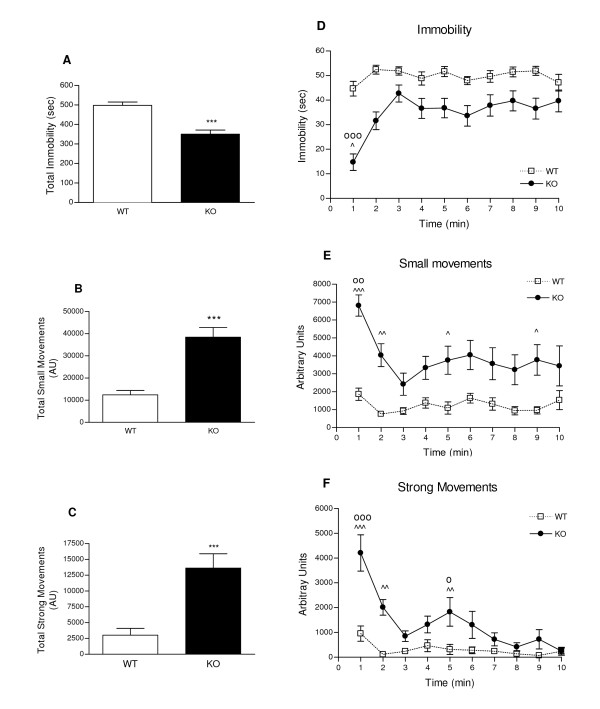
**Tail suspension test monitored for 10 min**. Sum of total scores of immobility are reported in sec (**2A**), small (**2B**) and strong movements (**2C**) in arbitrary units (AU). Time course of immobility (**2D**), small movements of running and body torsions (**2E**) and strong movements of body jerks and jumps (**2F**) are reported in bins of 1 min. *** P < 0.001 WT *vs*. KO, Students t-test; ^ P < 0.05, ^^ P < 0.01, ^^^ P <0.0001 WT *vs*. KO, Bonferroni *post hoc*; oo P < 0.01, ooo P < 0.001 *vs*. each different time point, Bonferroni *post hoc*; o P < 0.05 *vs*.7, 8, 9, 10 min, Bonferroni *post hoc*. WT n = 13, KO n = 13.

In the tail suspension test, WT and KO mice displayed significant score differences over the 10 min test period as shown in Figure 2. In Figure 2A, the overall immobility time of WT mice was of 83% of the total time; PKCI/HINT1 KO mice were 30% less immobile with an immobility time of 58% of the total time. Overall KO mice scores consisted of a 4 times higher frequency of small movements (Figure 2B) and 6 times higher frequency of strong movements of body jerks and jumps than the WT (Figure 2C). Time course analysis reveals an effect of time for the three parameters in a two-way ANOVA genotype × time, immobility F_(9,240) _= 4.34, P < 0.0001, small movements F_(9,240) _= 2.70, P = 0.0053 and strong movements F_(9,240) _= 9.54, P < 0.0001 (Figures 2D, 2E, 2F). In figure 2D, KO mice immobility can be viewed in two phases: one phase was the first minute of the test where initial immobility time is 25% of total time, and the second phase was a 9 min steady state where immobility increased to 63% of total time. Two similar phases can be observed with the production of small and strong movements, as illustrated in Figure 2E and Figure 2F, respectively. KO and WT mice behavior patterns were different, as indicated by the genotype effects: F_(1,240) _= 102.54, P < 0.0001 for immobility, F_(1,240) _= 93.57, P < 0.0001 for small movements, and F_(1,240) _= 57.01, P < 0.0001 for strong movements. Indeed, during the 10 min test WT mice exhibited a steady state behavior for each of the three parameters of the test (immobility, small and strong movements). KO mice demonstrated less immobility characterized by more energetic movements as illustrated by the overall measurement and the persistence of bouts of strong movements at 5 min of the test.

### Acute valproic acid decreases the activity of KO mice in the tail suspension test

**Figure 3:**
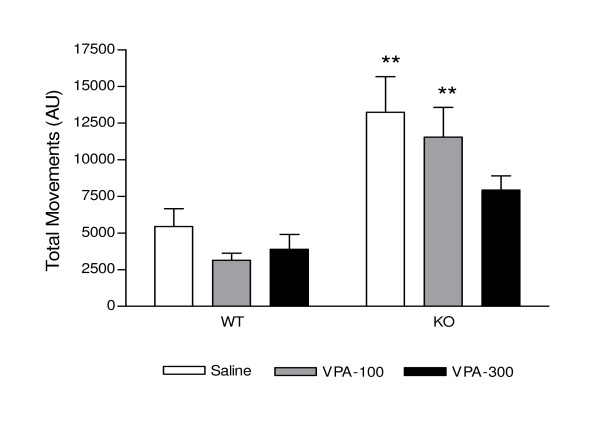
**Valproic acid effect on movements in the tail suspension test**. Small and strong movements in AU were measured 30 min following *ip *treatment with saline and valproic acid (VPA) 100 mg/kg and 300 mg/kg. Treatments were dispensed at 15 days intervals. ** P < 0.01 WT *vs*. KO, Bonferroni *post hoc*. WT n = 6, KO n = 8.

Figure 3 represents the effect of acute valproate treatment on total movements. A two-way ANOVA of genotype × treatment shows a genotype effect F_(1,33) _= 26.73, P < 0.0001 and no treatment effect F_(2,33) _= 2.33, P = 0.1135. Following saline injection, PKCI/HINT1 KO mice displayed 2.3 times more movements than the WT. Valproate (100 mg/kg or 300 mg/kg), when compared with saline treatment, did not induce any significant change in the number of movements in WT mice. PKCI/HINT1 KO mice pretreatment with valproate (300 mg/kg) revealed a statistically insignificant trend towards movement attenuation as compared with saline treatment. The number of movements reached was comparable to WT controls.

### In the light/dark test PKCI/HINT1 KO mice spent progressively more time in the light compartment than the WT

**Figure 4:**
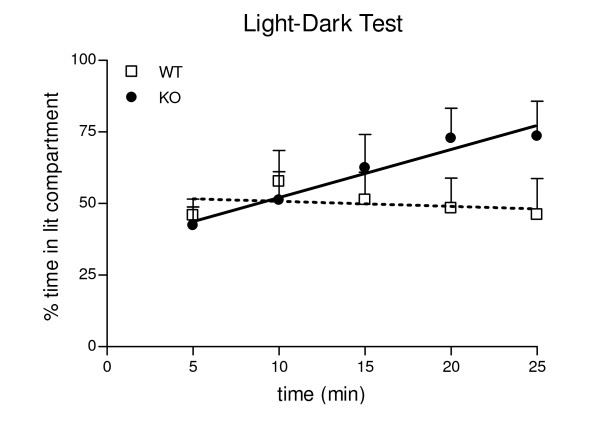
**Time course of the light/dark test**. Percent time spent in the lit compartment is represented within 5 min bins over the 25 min test period. Linear regressions: WT: y = -0.37 x + 52.6 (R^2 ^= 0.49); KO: y = 1.66 x + 36.98 (R^2 ^= 0.94). WT n = 9, KO n = 10.

Scores of the PKCI/HINT1 KO mice and their WT littermates in the light/dark test are presented in Figure 4 as a time course of the time spent in the lit compartment during the light-dark test. During the first 5 min of the test there was no difference between WT and KO mice; WT mice spent 47% of the time in the lit compartment and PKCI/HINT1 KO mice spent 42%. Curve analysis showed different slopes for WT and KO, F_(1,91) _= 5.41 P = 0.02. WT animals displayed a slight decrease in the time spent in lit compartment that reaches 41% at the end of the test with a negative linear regression coefficient of -0.37 not significantly different from zero, F_(1,43) _= 0.32 P = 0.57. PKCI/HINT1 KO mice spent progressively more time in the lit compartment to reach 75% at 25 min; this is illustrated by a positive linear regression coefficient of 1.68 which is significantly different from zero, F_(1,48) _= 7.93 P = 0.007. KO mice exhibited a significant progressive preference for the lit compartment.

### Place navigation performance is enhanced in the PKCI/HINT1 KO mice

**Figure 5:**
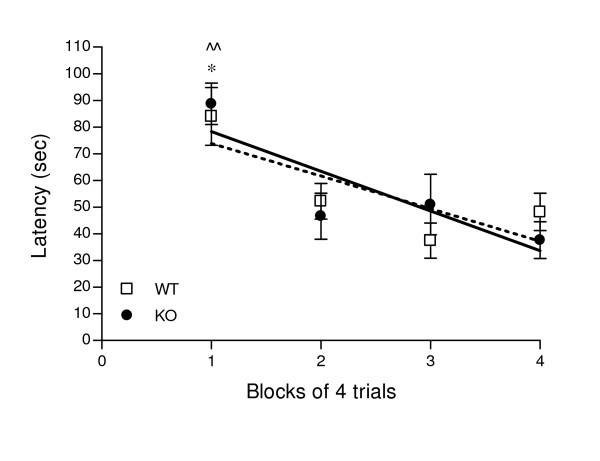
**Cued learning**. Learning performances in 16 trials of 2 min were assessed as latency to reach the beacon platform in the Morris water maze and are represented in 4 blocks of 4 trials. Linear regression curve of learning pattern for WT: y = -12.23 x + 86 and for KO: y = -15 x + 93 for KO. * at least P < 0.05 *vs*. each other trial for WT; ^^ at least P < 0.01 *vs*. each other trial for KO, Bonferroni *post hoc*. WT n = 7-10, KO n = 7-11.

The beacon cued learning test of the Morris water maze was used as a control experiment to assess ability to learn to swim to a cued goal that relies on intact eyesight, swimming ability, basic strategies of swimming away from the wall, climbing to the platform and motivation to escape from the water [38]. Performances, shown in Figure 5, were identical in WT and KO mice. Both animals learned to swim and escape to the platform with similar latencies.

**Figure 6:**
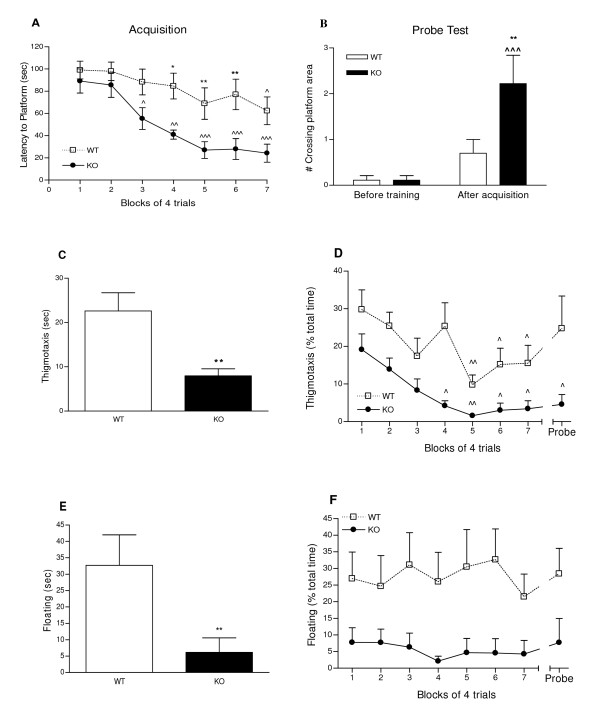
**Place navigation learning using proximal landmarks and memory**. **6A - Acquisition**. Learning performances in 28 trials of 2 min were measured as latency to reach the platform in sec, and are represented in blocks of 4 trials. ^P <0.05, ^^P < 0.01, ^^^P <0.001 *vs*. first block; * P < 0.05, **P < 0.01 WT *vs*. KO, Bonferroni *post hoc*. **6B - Probe test**. Retention was assessed as the number of the crossings of the platform area in a 1 min test before and after acquisition. ^^^ P < 0.001 *vs*. before acquisition; ** P < 0.01 WT *vs*. KO, Bonferroni *post hoc*. **Thigmotaxis **and **floating time **were measured during the acquisition phase and the subsequent probe test. Average thigmotaxis time in sec (**6C**). ** P < 0.01, WT *vs *KO, Student's t-test. Thigmotaxis time course as percent of total time (**6D**). ^ P < 0.05, ^^P < 0.01 *vs*. trial 1, Bonferroni *post hoc*. Average floating time in sec (**6E**). **P < 0.01 WT *vs *KO, Mann-Whitney. Floating time course as percent of total time (**6F**). WT n = 10, KO n = 10.

The Morris water maze version of a place navigation cognitive test depends on the function of the associative parietal cortex [39]. This test was chosen in relation to the anatomical localization of the decrease in PKCI/HINT1 in the dorsolateral prefrontal cortex of bipolar patients [32]. Performances of PKCI/HINT1 KO mice and WT littermates are presented in Figure 6. Latency to reach the platform within the 2 min test, plotted as a function of trials in Figure 6A, shows a trials effect, F_(6,126) _= 7.78, P < 0.0001 in a two-way ANOVA trials × genotype. This implies that both WT and KO animals underwent acquisition (learning). Initial latencies to reach the platform on the first trial were not different between WT and KO. However, a genotype effect, F_(1,126) _= 34.38 P < 0.0001, accounts for differences in the learning pattern on the trials. Significant acquisition was demonstrated in the last block of trials by WT mice with a latency of 62 sec, while PKCI/HINT1 KO mice displayed faster learning with a significantly lower latency of 55 sec as early as the third block of the trials. KO mice displayed significantly better learning, as attested by a 2.6 times lower latency to reach the platform on the last block of trials compared to the WT.

Retention (memory), shown in Figure 6B, was determined during a 60 sec probe test by counting the number of crossings of the platform area, both before training and 24 h following the last acquisition trial. Both WT and KO animals displayed an increase in scores after acquisition with trials effect, F_(1,34) _= 15.67 P = 0.0004, in a two-way ANOVA trials × genotype. Animals displayed differences in performances with a genotype effect, F_(1,34) _= 4.97 P = 0.0324. Before training, both WT and KO mice scores were near zero. After acquisition, WT animals crossed the platform area once whereas PKCI/HINT1 KO mice showed clear retention by crossing the platform area at least two times, which is considered criterion performance [40].

### Non cognitive factors are associated with learning and retention performances

Non cognitive factors like thigmotaxis and floating are often associated with performance in the Morris water maze [41]. Thigmotaxis time measured when mice swam at a distance within 10 cm of the wall of the pool is an indicator of open space anxiety-related behavior [42]. Figure 6C represents the overall thigmotaxis time during the acquisition phase and the probe test where the PKCI/HINT1 KO mice exhibited significantly 2.8 times less thigmotaxis than WT mice. Evolution of thigmotaxis with trials shown in Figure 6D reveals habituation during the acquisition phase with a trials effect, F_(7,144)_= 4.28, P = 0.0003, and a genotype effect, F_(1,144) _= 40.09, P < 0.0001, in a two-way ANOVA trials × genotype. When compared with trial 1, thigmotaxis significantly decreased at trials 5 and 4 for WT and KO respectively.

Floating time (*i.e*., immobility) was measured during the acquisition phase and the probe test. Figure 6E shows that overall floating scores for KO mice were significantly lower than WT by 5.3 times. The graph representing floating time as a function of trials in Figure 6F shows a genotype effect, F_(1,144) _= 39.03, P < 0.0001, in a two-way ANOVA trials × genotype, but no significant trials effect F_(7,144) _= 0.19 P = 0. 9879, the floating time for KO mice was lower than WT mice in every block. Thus WT and KO animals did not exhibit any change in floating time over the trials.

### PKCI/HINT1 KO mice basal hypothalamic-pituitary-adrenal axis activity is higher than in wild type mice

**Figure 7:**
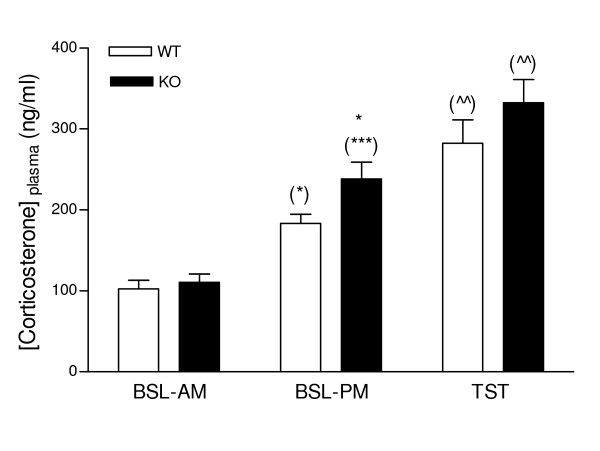
**Plasma corticosterone levelsin PKCI/HINT1 KO mice and their WT controls**. Plasma corticosterone levels (ng/ml of plasma) were determined for three conditions, as basal in the morning (BSL-AM), basal in the afternoon (BSL-PM), and following a 6 min tail suspension test performed in the afternoon (TST). * P < 0.05 WT *vs*. KO; (*) P < 0.05, (***) P < 0.001 *vs*. morning baseline; (^^) P < 0.01 *vs*. afternoon baseline, Bonferroni *post hoc*. WT n = 15, KO n = 15.

As shown in Figure 7, basal plasma corticosterone level in wild type animals was 102.5 ng/ml in the morning and increased by 79% to reach a value of 183.3 ng/ml in the afternoon. The acute stress of the tail suspension test induced an additional increase compared to the afternoon basal level. The same pattern of morning to afternoon variation and increased corticosterone levels after the stressor were observed in the PKCI/HINT1 KO mice, F_(2,84) _= 49.8, P < 0.0001, for condition in a 2-way ANOVA condition × genotype. Additionally, KO mice displayed an afternoon baseline level that was 30% higher than the WT mice, F_(1,84) _= 5.27, P = 0.0243 for genotype in a 2-way ANOVA condition × genotype. Immediately following a tail suspension test, WT and KO mice displayed comparable responses to stress, with increased of corticosterone levels reaching 282 ng/ml and 332 ng/ml respectively.

## Discussion

We searched for a distinctive phenotype of PKCI/HINT1 KO mice in order to determine the putative role of the PKCI/HINT1 protein in brain function and in particular its involvement in mood regulation.

In order to assess antidepressant-like behavior, we used two standard tests, the Porsolt's forced swim test and the tail suspension test. In both tests PKCI/HINT1 KO mice exhibited less immobility than their WT littermates. This phenotype was further confirmed in the Morris water maze where KO animals exhibited less floating time than the WT. Decreased immobility in the FST and the TST may be due to an antidepressant or a general stimulatory effect, as pointed out by pharmacological studies; antidepressant effects are manifested in attempts to escape, while stimulants lead to motor stimulation that can be measured as an increase in spontaneous activity in the open field test [3,43]. As PKCI/HINT1 KO mice did not display spontaneous hyperactivity in the open field test [33], the decreases in immobility presented here are likely due to persistence in attempts to escape rather than generalized motor stimulation. This is exemplified in the persistence of small and strong movements in the TST.

Treatment with the mood stabilizer valproate at 300 mg/kg tended to attenuate the movements of KO mice. However a non-significant trend of decreased movements in the WT mice treated with 100 or 300 mg/kg suggests a possible sedative effect of the drug in stead of mood regulation, which could be specifically assessed in the open field test.

We used the light-dark test based on the mouse's natural preference for the dark and avoidance of bright spaces to assess anxiety-related behavior. In the first 5 min corresponding to the classical version of the test that assesses light/dark preference, there was no difference between KO and the WT controls, as both animal types spent an average of 45% of the total time in the lit compartment. A similar slight preference for the dark compartment was reported in mice from the C57Bl and 129/SveV strains that were naïve to any test when submitted to the light/dark box [44]. The time course of the 25 min test showed that time spent in the lit compartment increased progressively in PKCI/HINT1 KO mice, so that in the last 5 min KO mice spent significantly more time in the lit compartment than WT mice. This phenotype could not be totally attributed to an anxiolytic effect and the 25 min light/dark test would require pharmacological validation to be related to anxiety. An alternative explanation would be that, by showing a preference for the naturally aversive properties of the light spaces, the KO mice were exhibiting a risk-taking behavior. Additionally, PKCI/HINT1 KO mice in the Morris water maze place navigation task displayed significantly less thigmotaxis than the WT mice, showing less anxiety behavior related to open space. In that case, thigmotaxis might have some influence on the learning performances. However, as in the beacon cued learning test with a visible escape WT and KO mice showed similar learning performances, it is probable that in our mice thigmotaxis in the water maze is related to the higher anxiogenic conditions of non visible escape in the place navigation task.

In several experiments presented here, habituation was an important factor, such as habituation to an aversive or novel stimulus. Thus some of the results implicated here may imply that there is accelerated habituation in PKCI/HINT1 KO mice, at least to the anxiety-provoking aspects of the experimental environments. Perhaps due to this difference, PKCI/HINT1 KO mice exhibited a faster acquisition in the place navigation learning task, and consequently better retention.

By contrast in the modified version of the FST, repeated testing increased immobility to a greater degree in WT mice than in PKCI/HINT1 KO mice. On the second day of FST testing, an increased immobility was observed in WT controls compared to the first trial, while PKCI/HINT1 KO mice remained as active as they were during the first exposure day, not demonstrating increased immobility until the last test of the second day. In the FST paradigm, increased immobility can potentially be influenced by several factors. Although generally considered to measure behavioral despair in relation to the effect of anti-depressant drugs [5] it can also reflect learned habituation to an environment that has become familiar to the animal thereby including a memory component on the second exposure. Lastly, it could reflect a relatively successful coping strategy that employs energy-conserving behavior and may confer a survival advantage (for review [45]).

As KO mice displayed higher performances in the learning task of place navigation, it is less likely that less learning capacity is responsible for their delayed increased immobility in the FST. Yet, PKCI/HINT1 KO mice's persistent struggling in both FST and TST could be related to less coping behavior [45], and thus express excessive goal pursuit as previously suggested in the FST [46]. In the same sense, it is also noteworthy that in the goal-directed task of cocaine-induced conditioned place preference, PKCI/HINT1 KO mice displayed enhanced performances as compared with WT (manuscript in preparation).

Thus the current behavioral data indicate that PKCI/HINT1 has an important influence on depression and open space-anxiety related tests. Moreover, PKCI/HINT1 plays an important role in learning and exploration in the place navigation water maze and the development of preference for aversive environment in the 25 min version of the light/dark test.

Parallel to the behavioral phenotypes, we assessed the activity of the HPA axis *via *the measurement of plasma corticosterone levels. Noticeably, in the same time frame corresponding to the behavioral experiments, PKCI/HINT1 KO mice displayed a higher afternoon basal plasma corticosterone level than the WT controls.

However, the elevated level of corticosterone measured in PKCI/HINT1 KO mice is actually the opposite of what would be expected given the observed behavioral changes. Antidepressant in FST and TST and anxiolytic-like behaviors in the light/dark test are usually associated with decreased plasma corticosterone levels [25]. On the contrary, in the laboratory and clinically, increased plasma corticosterone/cortisol is associated with increased depression-related and anxiety-like behaviors [17,21-23,47-49]. However, the phenotypes of antidepressant-like and less anxiety-related behaviors were associated with increased corticosterone levels in PKCI/HINT1 KO mice. This has an interesting parallel with clinical observations of increased plasma cortisol levels in manic patients which was not an effect of stress but was described as a feature of the disease [18]. Indeed, HPA axis dysfunction was reported in bipolar disorder patients [50,51] and in the mixed mania subtype of bipolar disorder patients [19]. In all cases, higher basal plasma cortisol levels were found in bipolar patients relative to the controls. Higher plasma cortisol levels in manic patients, compared with healthy controls, were in the same proportional range as what we observed in the PKCI/HINT1 mice [18]. Moreover, in bipolar patients, the difference occurred in the samples collected in the morning, which corresponds to the awaking time [19,51]. This phenomenon coincides with the findings in our mice. The difference in corticosterone levels between KO and WT mice occurred during the window time that also corresponds to awaking time in these nocturnal animals. When tested for TST and FST in the morning between 9:00 to 10:00 am, WT and PKCI/HINT1 KO mice displayed the same amount of immobility time (data not shown).

The difference in plasma corticosterone levels should not be attributed to a stress response due to handling since we did not see any difference between WT and KO in the morning samples. In WT mice, morning and afternoon basal corticosterone levels were similar to those previously reported [21,52]. Moreover, WT and KO mice presented similar plasma corticosterone responses to the acute stress of tail suspension. Those responses were in the same range as the one described as the maximum response to stress measured following a restraint stress in mice, *ca *300 ng/ml [52]. Accordingly, the afternoon-enhanced basal corticosterone levels may be considered an endocrine feature specific to the phenotype of PKCI/HINT1 KO mice.

Additionally, in KO mice, hyperactivity in the tail suspension test tended to be attenuated by acute treatment with the clinically effective anti-mania drug valproic acid, suggesting a facet of mania-like behavior. Therefore, the afternoon behavioral and endocrine phenotypes of PKCI/HINT1 KO mice could be analogous to mania symptoms associated with high plasma cortisol [9,14,53-57]. In that regard, enhanced performances in the place navigation task and persistent struggling in the TST could be considered as enhanced goal-directed activity associated with enhanced energy. Moreover these data are in consistent with microarray results demonstrating a decrease in PKCI/HINT1 expression in the brains of bipolar disorder patients, making it a candidate molecule for the disease [32]. Thus our results may suggest that the PKCI/HINT1 gene may be important in the neuropathology of mania.

Our speculations on our results are based on attempting to draw parallels between our behavioral, endocrine and pharmacological observations and clinical descriptions of disease. One may argue that this extrapolation from mice to human patients is excessive specifically because there is no current valid rodent model for studying mania [58]. Further study of the function of PKCI/HINT1 in the nervous system could help in outlining a theoretical validation. Originally PKCI/HINT1 isolated from bovine brain extracts was identified as an *in vitro *inhibitor of PKC isoforms [59] and PKC inhibitors are promising targets for bipolar disorder drug development [60]. However, the physiological significance of PKCI/HINT1's ability to inhibit PKC has been questioned [59] and so far its exact biochemical function is unclear. In our study, although the exact role of PKCI/HINT1 in the development of those combined phenotypes is unknown, some speculations can be outlined based on the modulation by the protein of the mu opioid receptor (MOPr). PKCI/HINT1 regulates the MOPr by suppressing its desensitization and its PKC-induced phosphorylation [61]. A deficiency in the expression of PKCI/HINT1 in mice can significantly enhance both basal and morphine-induced analgesia [61,62]. Interestingly, in one study, manic patients exhibited higher analgesia to experimentally-induced pain than healthy matching controls [63]. Also, opioid analgesics can precipitate a hypomanic/manic reaction in a significant percentage of patients with bipolar disorder while having an antidepressant effect in others [64]. In acutely manic patients, methadone decreased symptoms of euphoria, elation, and grandiosity, and significantly decreased plasma cortisol levels. This implies a potentially important interrelationship between the central endogenous opioid peptide systems, neuroendocrine regulatory factors, and the pathophysiology of affective disorder [65]. This could be linked to the effects of MOPr activity on the HPA function in humans where stimulation of the MOPr by the specific agonist fentanyl decreases the level of plasma cortisol [66] and morning MOPr blockade by naloxone induces an increase in ACTH and plasma cortisol levels [67]. This could be a mechanism through which changes in PKCI/HINT1 interactions with MOPr modulate MOPr-associated functions.

## Conclusion

PKCI/HINT1 KO mice displayed a phenotype of behavioral endocrine features indicative of changes of mood function, including anxiolytic-like, antidepressant-like behaviors that tend to be blocked by the mood stabilizer valproate, in conjunction with elevated levels of plasma corticosterone. These results suggest that the PKCI/HINT 1 gene could be important for the mood regulation function in the CNS.

## Methods

### PKCI/HINT1 KO mice

The generation of PKCI/HINT1 KO mice was described previously; 96% of the genetic background of these mice and their WT littermates is from the 129SvJ strain [68]. In the present study, KO and WT animals were derived from heterozygous PKCI/HINT1 ^+/- ^breeding pairs. The genotype of all progeny was confirmed by PCR analysis of DNA extracted from tail biopsies. Animals were housed 4-5/cage and maintained under standard laboratory conditions with food and water provided *ad libitum*. Male animals were tested between 6 to 9 months of age. Wild type and PKCI/HINT1 KO groups were matched for age in all experiments. Animals were submitted to a 12 hours dark/light cycle with light on at 7:00 am, and experiments were performed during the light phase. For each experiment, animals were brought into the experimental room 30 min prior to the experiment in order for them to acclimate to the environment. Each experiment was performed independently using a new group of animals. All studies were conducted with a protocol approved by the University of Maryland, School of Pharmacy IACUC, and studies conformed to the NIH Guidelines for the Care and Use of Laboratory Animals.

### Forced swim test

The test was based on the original version of the forced swim test of Porsolt for mice [69] with modification; scoring was performed in real time by observers unaware of the genotype of the mice. Mice were placed in a 5 l cylinder (40 cm high, 25 cm diameter) filled with 3.5 l of water, where they swam without the ability to touch the bottom; water temperature was set at 30 ± 1°C to avoid severe hypothermia [70]. The total time that the mice spent immobile was measured, using a stopwatch. Immobility was determined when the mouse was only making movements necessary to keep its head above the water and maintained a stationary posture; a stopwatch was started within the first 2 sec immobility was observed. In this posture forelimbs of the mouse are motionless and directed forward, the tail is directed outward and the hind legs are in limited motion. No animals showed difficulty in swimming or in staying afloat. We used a 2 day procedure as previously described [71]. On day 1, the mice were placed in water to swim for a single trial of 15 min, and immobility was recorded during the last 4 min of the trial. Day 2 was aimed at assessing the learning and habituation components of the repeated test. Mice were placed in water through a series of four trials of 6 min each and immobility was recorded during the last 4 min of each trial. Each trial was followed by an 8 min rest period when the animals were dried with towels and returned to their cages; the water in the cylinder was changed between each subject. Experiments were performed between 2:00 and 4:00 pm.

### Tail suspension test

The automated tail suspension test (Med Associates, St Albans VT) as previously described [72,73] was used to assess antidepressant-like behavior. The haemodynamic stress of being hung in an uncontrollable fashion by the tail causes the animals to engage in three types of escape-oriented movements: (1) running movements forward or backwards; (2) body torsions with attempts to catch the suspended body; and (3) body jerks followed temporally by bouts of immobility [4,43]. A mouse is considered immobile when it either hangs passively and completely motionless, does not move its paws, or there is an absence of initiated movements.

The automated device (Med Associates, St Albans VT) consists of a box (box size: 32 × 33 × 33 cm) that is open on the front side with a vertical aluminum bar (bar size: 11.5 × 2.2 × 0.15 cm), suspended from the top, connected to a strain gauge that detects any movements from the mouse. Mice were suspended by the tail and secured with a tape for a 10 min test. The base of their tail was aligned with the bottom of the bar. The total duration of immobility was calculated as the time when the force of the mouse's movements was below a preset lower threshold. An optimal lower threshold was determined by comparing scores of immobility rated manually with scores from the automated device in preliminary studies. Immobility was defined as the absence of initiated movements and included passive swaying. An upper threshold was determined in order to detect only vigorous movements. Thus the following settings were used in all experiments: lower threshold = 5, upper threshold = 50, gain = 4, time constant = 0.25, resolution = 100 msec. The automatic equipment monitored the duration of immobility as the time below lower threshold. The vigor of activity was quantified as small movements, corresponding to running movements and body torsions between lower and upper threshold and strong movements of jerks and jumps rated above the upper threshold.

Experiments were performed between 2:00 and 4:00 pm.

### Drug treatment

Valproic acid as sodium salt (Alexis Biochemicals, USA) was prepared freshly in saline (NaCl 0.9%) and was injected intraperitoneally (*ip*) at a volume of 10 ml/kg of body weight, 30 min before the TST at doses of 100 mg/kg or 300 mg/kg.

### Light-dark test

The light-dark test to assess bright-space related anxiety [74] was conducted in a dark-light insert consisting of two compartments of same dimensions (13.95 cm × 27.9 cm) separated with a transparent wall pierced with an open door. The black compartment had opaque black walls and roof while the lit compartment was entirely transparent to light. The insert was placed into an open field chamber equipped with 16 infra-red beams allowing tracking of the animal position. The test was automated using activity monitor software (Med Associates, St Albans VT). Mice were placed in the box for a total time of 25 min; the amount of time spent by the animal in each compartment was monitored by 5 periods of 5 min each and reported in seconds. The first 5 min bin was to assess anxiety-related behavior while the remaining 20 min assessed putative preference development. Experiments were performed between 2:00 and 4:00 pm. Results are expressed as mean ± SEM of time spent in the lit compartment during each 5 min bin.

### Morris water maze

The Morris water maze experiment consists of two tests: cued learning [38] and place navigation using proximal landmarks [39]. The two tests were performed independently using separate groups of animals. The maze consisted of a 122 cm diameter circular pool of 25 cm height sides filled with 25 ± 1°C water and made opaque with milk. It was oriented in relation to the cardinal points, N, S, E, W. The only mean of escape was through a circular platform of 9 cm diameter and 11 cm height submerged 0.5 cm below the surface.

**For the cued learning test**, the platform was kept invisible but was beacon cued with a striped ruler emerging at 12 cm above the surface of the water so that the mice could locate it directly by sight. The test consisted of 4 trials of 2 min per day for 4 days. It assessed the animal's motivation and ability to swim to escape from the aversive situation of being placed into the water by associating the beacon-cued platform with the escape. For each trial the platform was located at the center of one of four quadrants NE, SE, NW, SW; the mouse entered the pool, facing the wall, from a different starting point each time so that the direct route to the platform differed each time. Cued learning was measured as the latency to reach the platform (sec). Mice that failed to reach the platform within the 2 min time limit were ascribed an escape latency of 120 sec and guided to the platform. Mice were allowed to stay on the platform for 5 sec before being dried with towels and returned to their home cages.

**To assess place navigation **using proximal landmarks for navigation, the pool was surrounded by white curtains to isolate it from distal landmarks. Three unscalable objects placed directly inside of the pool and emerging from the surface of the water by 8 cm served as proximal landmarks for the invisible platform. The objects were 500 ml square bottles (side: 7 cm, height: 18 cm) filled up with white sand. The bottles were distinguishable from each other by the color of the caps (solid navy blue, white with black stripes, or white with black dots) and by the pattern of the protruding portion (white, white with black vertical stripes, or white with black dots). The test started with an initial session training consisting of 2 trials separated by 30 min interval; mice were placed on the platform for 15 sec to observe the landmarks prior to being put in the water for 2 min. If the mouse did not find the platform within the allotted time, it was guided to it and was allowed to remain on it for 5 sec before being dried with towels and returned to its home cage. The learning phase started one day following the initial training. It consisted of 4 trials of 2 min per day for 7 days; the interval between each trial was at least 30 min. Before each trial both the objects and the platform were rotated 120° with respect to the center of the pool in a pseudorandom direction; mice entered the pool, facing the wall, from different starting positions that were randomly defined. Acquisition was assessed through the latency to reach the platform (sec). Mice that failed to reach the platform within the 2 min time limit were ascribed an escape latency of 120 sec and guided to the platform. Mice were allowed to stay on the platform for 5 sec before being dried with towels and returned to their home cages. One day following the last acquisition trial, retention was evaluated in a probe trial of 60 sec during which the platform was removed and the number of times crossing the former platform area was measured. For probe tests a leaden object the same dimension of the platform was placed on the bottom of the pool to facilitate previous platform location. Trials were video recorded using a digital camera placed above the pool.

### Corticosterone level measurement

To determine plasmatic corticosterone levels in mice, 100 to 200 microliters of blood were collected from retro-orbital bleeding (n = 15 mice in each group) into heparin (1000 U/ml Sigma-Aldrich #H3393) pre-coated microcentrifuge tubes, centrifuged at 2500 × g for 5 min at room temperature; the plasma supernatant was stored at -70°C until corticosterone measurement. Corticosterone levels were measured within 2 weeks following blood collection by radioimmunoassay using a Corticosterone kit (MP Biomedical # 07120002) according to the instructions of the manufacturer. Morning and afternoon basal levels were determined from blood collected in the morning 2 to 3 hours after light onset, between 9:00-10:00 am, and in the afternoon 2 to 3 hours before the light was turned off, between 4:00-5:00 pm. Plasma corticosterone levels following acute stress were also determined from blood collected immediately after a 6 min tail suspension test performed in the afternoon in the same time window of 4:00 to 5:00 pm.

### Statistics

For all data, normality was assessed using the Kolmogorov-Smirnoff test. Scores of behavioral experiments test were analyzed using a two way ANOVA and Bonferroni *post hoc *test for comparison between groups when significant (p < 0.05). Simple comparison between 2 groups was performed using Student's t-test for normal groups and the Mann-Whitney test otherwise. For plasma corticosterone levels, data were analyzed using a 2 way ANOVA genotype × conditions, and a Bonferroni *post hoc *test for comparison between groups when significant (p < 0.05). Results are represented as mean ± standard error of the mean (SEM) in the figures. Analyses were performed using Graphpad Prism version 4.00 for windows (Graphpad Software, San Diego, CA, USA).

## Competing interests

"Novel neurological function of mPKCI" is the object of an eponymous international patent under the publication number: WO 2007/092598.

## Authors' contributions

EB participated in the conception and the design of the study, carried out the experiments, performed the analyses of data and wrote the manuscript. JBW participated in the conception and the design of the study, and the manuscript writing. All authors read and approved the final manuscript.
